# A Connector for Integrating NGSI-LD Data into Open Data Portals

**DOI:** 10.3390/s24051695

**Published:** 2024-03-06

**Authors:** Laura Martín, Jorge Lanza, Víctor González, Juan Ramón Santana, Pablo Sotres, Luis Sánchez

**Affiliations:** Network Planning and Mobile Communications Laboratory, Universidad de Cantabria, Plaza de la Ciencia s/n, 39005 Santander, Spain; jlanza@tlmat.unican.es (J.L.); vgonzalez@tlmat.unican.es (V.G.); jrsantana@tlmat.unican.es (J.R.S.); psotres@tlmat.unican.es (P.S.)

**Keywords:** NGSI-LD, Open Data, CKAN, DCAT-AP, interworking

## Abstract

Nowadays, there are plenty of data sources generating massive amounts of information that, combined with novel data analytics frameworks, are meant to support optimisation in many application domains. Nonetheless, there are still shortcomings in terms of data discoverability, accessibility and interoperability. Open Data portals have emerged as a shift towards openness and discoverability. However, they do not impose any condition to the data itself, just stipulate how datasets have to be described. Alternatively, the NGSI-LD standard pursues harmonisation in terms of data modelling and accessibility. This paper presents a solution that bridges these two domains (i.e., Open Data portals and NGSI-LD-based data) in order to keep benefiting from the structured description of datasets offered by Open Data portals, while ensuring the interoperability provided by the NGSI-LD standard. Our solution aggregates the data into coherent datasets and generate high-quality descriptions, ensuring comprehensiveness, interoperability and accessibility. The proposed solution has been validated through a real-world implementation that exposes IoT data in NGSI-LD format through the European Data Portal (EDP). Moreover, the results from the Metadata Quality Assessment that the EDP implements, show that the datasets’ descriptions generated achieve excellent ranking in terms of the Findability, Accessibility, Interoperability and Reusability (FAIR) data principles.

## 1. Introduction

Proliferation of data sources is creating an abundance of information that is called to bring benefits for both the private and public sectors, increasing, for example, administrations’ transparency and availability or fostering efficiency of public services [1]. The influence of Information and Communication Technology (ICT) on public values, particularly in bolstering transparency, has been acknowledged since the inception of computers in public agencies and institutions. This trend gained momentum with the widespread availability of Internet access in developed countries, intensifying competition in information supply and significantly easing the dissemination of information.

In the contemporary landscape, global technological advancements heavily rely on data, and the utilisation of ICT in the public sector offers multifaceted benefits. Since 2010, the concept of Open Data has been intricately linked to broader open government reforms, creating a connection between Open Data and reforms in public management [2]. For example, Open Government Data has become a pivotal concept guiding government initiatives to enhance transparency and accountability regarding the utilisation of public resources. However, the Open Data movement goes much further than openly disclosing data from public sectors. It has emerged as a catalyst for making data accessible to the public on the Internet. Increasingly, data from governmental, public, but also private entities is being released online, primarily through Open Data portals. This extends to various sectors and contributes to the progression toward smart cities [3].

Nonetheless, as the volume of published resources grows, concerns arise regarding the quality and interoperability of the data sources and their corresponding metadata. These issues pose challenges to the usability of the resources. The main software frameworks used for this kind of portals, like Comprehensive Knowledge Archive Network (CKAN), Socrata or OpenDataSoft, focus on the description of the datasets available through the portal, this is, on the so-called metadata, while the actual data does not have to comply with any specific standard. It is usual that, even within the same portal, different datasets containing the same kind of data are hardly interoperable as they do not follow any specific information or data model. This situation gets worse the more inter-domain data the Open Data Portal stores. For example, the European Data Portal (EDP) that harvests data from other portals all over Europe, or National Data Portals that gather data from different Regional or Municipal Governments.

This is the main limitation that the work that is described in this paper is tackling. It is critical that the data that can be retrieved through Open Data portals adheres to standard information and data models so that, besides openly discoverable and accessible, it is actually usable and re-usable, without facing tough interoperability challenges. Open Data portals, and the technologies on which they are based, focus on the metadata, specifically on the datasets’ descriptions leaving the features of the actual data totally up to the data provider.

In this sense, heterogeneity in data access stands as a significant obstacle impeding the broad usability of data, hindering the implementation of solutions that depend on data from various sources. Hence, ensuring data interoperability, encompassing both access and modelling, becomes imperative to facilitate the development of adaptable and reproducible solutions. To achieve data interoperability, consensus is required on the technological interfaces and data modelling employed in data exchange.

In this context, the Next Generation Service Interface with Linked Data (NGSI-LD) standard emerges as a potential solution to harmonise data access specifications, promoting interoperability among different data providers and consumers. NGSI-LD, an European Telecommunications Standards Institute (ETSI) standard, offers a comprehensive specification for enabling context data management. It can enhance access to context information by defining an Application Programming Interface (API) [4] and an information model [5] for diverse participants. This standard is the core interface of the FIWARE open-source ecosystem. NGSI-LD is already in use in numerous real-world pilots [6,7], providing a flexible and reliable means to address the challenges of data interoperability in scenarios where harmonising access to data from heterogeneous sources is essential.

However, the opposite situation to the one described above as the main limitation addressed by our work is faced in case the solution is solely based on NGSI-LD. Its API can be employed by applications to consume data, but it does not offer native tools or frameworks to promote discoverability of the datasets that are accessible through such API. Thus, NGSI-LD, on its own, cannot be the sole solution in terms of providing the Findability, Accessibility, Interoperability and Reusability (FAIR) data principles.

The main novelty that we are bringing with the work that is described in the paper is to bring together the abovementioned two technologies, namely, CKAN-based Open Data portals employing Data Catalog Vocabulary-Application Profile (DCAT-AP) specification for describing their datasets, and NGSI-LD-based data, so that they can mutually cover each other limitations and realise a solution maximizing the Findability, Accessibility, Interoperability and Reusability (Findability, Accessibility, Interoperability and Reusability (FAIR)) data principles. The proposed solution eases the process for data providers managing their data following the NGSI-LD standard to generate the corresponding metadata (i.e., datasets’ description) that is necessary to automatically expose their data on a CKAN-based Open Data portals. Considering the great significance that FAIR principles have been acquiring in the framework of data sharing, developing a solution that contributes to the maximization of such principles paves the way for establishing larger critical mass around well-established data and metadata modelling technologies (i.e., NGSI-LD and DCAT-AP, respectively) that should help in harmonizing the nowadays barely interoperable data exchange ecosystem. To the best of our knowledge there are no similar alternatives proposed in the literature.

In this paper, we are promoting the synergies that can be established among the Open Data portals, in particular those based on CKAN using the DCAT-AP specification for describing the datasets that they contain, for data availability and discoverability, and the NGSI-LD standard for data interoperability. In this sense, this article is presenting the design and development of the solution, comprising a set of connectors that bridge these two domains. This solution is able to render NGSI-LD data accessible through Open Data portals by aggregating the data into coherent datasets and generate high-quality descriptions, ensuring comprehensiveness, interoperability and accessibility.

The key idea is to integrate the capacity that Open Data portals have to describe the datasets as a whole, by the use of the well-established World Wide Web Consortium (W3C) DCAT-AP specification [8], and the ability of providing the actual data (i.e., the data items within the available datasets) using a standard, interoperable and extendable information model through the adoption of NGSI-LD syntax and semantics. This integration not only enriches the publicly available information but also expands the potential for insightful analysis and informed decision-making, aligning the worlds of IoT-generated data and open public repositories.

The remaining of the paper is structured as follows. In Section 2 a brief review of key aspects of NGSI-LD and Open Data is made, together with a discussion on related works currently available in the literature. Section 3 showcases the binding among the several data formats used throughout the architecture. The NGSI-LD-to-CKAN connector design and implementation details are described in Section 4. In Section 5 the practical validation that has been carried out to assess the behaviour and resulting integration is presented. Finally, Section 6 concludes the paper highlighting the main contributions that the work described in this paper is bringing into the existing data management and distribution ecosystem.

## 2. Background

Before diving into the proposed technical solution, it is crucial to review the key concepts around which this solution has been developed. Firstly, introducing the Open Data paradigm, followed by the well-known Open Data system CKAN, and concluding with the widely used DCAT-AP specification. Afterwards, the NGSI-LD standard main features are described, focusing on the information model itself, the Smart Data Models initiative and the API for accessing data from compliant Context Brokers. Moreover, a review of related works is also provided in this section.

### 2.1. Open Data Portals

Since the introduction of the first open data portals by the United States government in 2009 and the United Kingdom in 2010, many other countries and organisations have initiated similar open data projects and launched data portals. These initiatives aim to facilitate public access to a diverse array of data available in various formats and spanning a wide range of domains [9]. This trend aligns with the observations reported in [10], which noted a continuous growth in the number of datasets and sources.

Numerous countries, including a substantial number of EU Member States, have embraced this trend, with some local governments (e.g., city governments) also participating [11]. Many of these portals utilise the CKAN, a free and open-source data portal platform developed and maintained by Open Knowledge. Consequently, they possess a robust standard API, opening up the possibility of amalgamating their catalogues to establish a unified global entry point for discovering and utilising government data. Furthermore, in line with its open-source approach, CKAN allows the development of extensions that modify or add different functionalities to the default Data Portal. This has led to the existence of extensions developed by the CKAN community [12].

As for the representation and sharing of data descriptions, the DCAT-AP specification is the most widely used. DCAT-AP is a Data Catalog Vocabulary (DCAT) profile that provides a standardised way to describe Catalogues containing Datasets and Data Services. It is also designed to enhance interoperability and facilitate the exchange of metadata across different data portals and catalog systems. The standard includes elements such as dataset titles, descriptions, keywords, and distribution information to ensure comprehensive and harmonised metadata representation [8].

### 2.2. NGSI-LD

The information model specified by the NGSI-LD standard is designed to facilitate interoperability between various entities in a digital ecosystem. The Context Broker (CB) is the key component in the NGSI-LD architecture. It is responsible for managing context information. Furthermore, it enables temporal queries by providing persistent storage of NGSI-LD data. Several architectures are possible depending on the organisation of different CBs, with federation being one of the most commonly adopted. The federated architecture provides the system with a single point of access to the data through the so-called Federated Context Broker (FCB). This FCB has different CBs registered underneath it. As such, any request made to the FCB via this single point of access is forwarded to its federated CBs and the results obtained are aggregated to be provided back to the consumer (i.e., the one that made the request). Hence, as noted above, the underlying architecture is hidden from the consumer, exposing only the FCB API [4].

NGSI-LD standard only specifies an abstract information model. For the actual modelling of specific-domain context, the Smart Data Models initiative provides a common framework for modelling and describing data entities, attributes and relationships to enable the creation of a digital marketplace of interoperable and replicable smart solutions across multiple sectors. This initiative provides more than 900 NGSI-LD compliant data models for describing elements in the Smart Agriculture, Smart Cities and Smart Environment domains, among others, and can be considered a de facto standard for information representation in these areas [13].

### 2.3. Related Works

Open data portals today play the role of an interface that creates transparency [14]. However, to provide these opportunities, open data portals should offer users a wide range of mechanisms to enable them to effectively discover, extract and use data [15]. Nonetheless, according to [16], greater attention needs to be paid to what the transparency promoted by ICT is and how it can be achieved. As it is introduced in [17] the actual potential of Open Data is not on the release of any information asset as it creates a modern, electronic “Tower of Babel” based on incompatible or poorly compatible catalogues through which multiple agents release information.

Data service is a critical component for Open Data which guarantees the availability of data to users in the form of structured and machine-readable open datasets. Though, aspects such as usability, quality, and interoperability should be considered in building such open datasets. The diversity of datasets usually hampers unlocking the full potential value of data. Interoperability addresses the ability of open data platforms and data services to communicate, exchange and consume data, and to operate effectively together.

Therefore, various studies have attempted to systematically delineate different interoperability layers. For example, the European Interoperability Framework (EIF) [18], initiated by the European Commission to promote seamless service interoperability and data flows across European public administrations, identifies interoperability layers (Technical, Semantic, Organizational, and Legal). Drawing inspiration from the EIF, the European Open Science Cloud (EOSC) Interoperability Framework [19] incorporates these layers to enhance interoperability in the research and science domain, aligning with FAIR principles (Findability, Accessibility, Interoperability, and Reusability) for scientific data management [20]. It’s worth noting that while EIF provides conceptual modelling, EOSC concentrates on research data exchange, lacking the flexibility required for the multifaceted nature of versatile Open Data portals.

In a different approach, a framework for federated interoperability proposed by [21] integrates graph theory and Model-Driven Engineering to enable dynamic data transformation and integration among heterogeneous relational database systems. In detail, their proposed solution initially explores original relational databases to identify source and target data models, creating their corresponding graph representations. Subsequently, it computes the similarity between elements (nodes and edges) in the two graphs to generate a set of transformation rules for mapping source data to the target data structure. This work serves as a preliminary step in aligning existing datasets with the NGSI-LD standard information model, enhancing data exploitability by standardising datasets according to a common model, as proposed in this paper.

In [22] authors identified several problems on the CKAN-based Open Data portals ranging from data management limitation, to no real-time features or absence of interconnection standard, and proposes a solution for improving interconnectivity and data usability expanding the CKAN services. However, it still focuses on the harmonisation of datasets metadata rather than on addressing the challenge of contributing to the usability of the data itself.

The Open Data Development Model (ODDM) proposed in [23] was meant to allow building a platform for interdisciplinary research that makes it easy to extract value from the open data by integrating open data from various sources. However, it is again restricted to the theoretical modelling for developing interoperable Open Data portals, but without a tangible implementation based on well-established standards and best-practices as we are proposing in this work.

## 3. Data Modelling Analysis

The key role of the proposed connector is to accomplish data interpretation and adaptation between both domains (i.e., the context data represented as NGSI-LD entities, and the CKAN datasets containing such context data, which are described using DCAT-AP). In order to understand the modules that have been specified for making the two domains to interact with each other, it is crucial to understand the relation between the different data models’ features. Table 1, Table 2 and Table 3 present the bindings between concepts belonging to the different domains involved in the solution that have been elicited after the analysis of the information models used in those domains.

Identifying the data to be represented was the preliminary step in the analysis. In this architecture, the information that flows among the components is not the data points themselves, but rather the description of datasets. In other words, descriptive metadata about a collection of data. As mentioned in Section 2.1, DCAT-AP is the reference specification for the exchange of information about catalogues of datasets and data services. Hence, in our solution we adopted it as a baseline data model.

Since the cornerstone of the architecture lies in the NGSI-LD environment, the data models belonging to the DCAT-AP subject [24] of the Smart Data Models initiative, which adapt this specification to the NGSI-LD context, have been used. In particular, the Catalogue, Dataset and Distribution models, which directly adapt these terms from the DCAT-AP v2.1.1 specification [8].

The next data format found in the architecture is the CKAN format. It consists of JavaScript Object Notation (JSON) objects with specific structures and properties according to the defined term. The terms Catalogue, Dataset and Distribution, which are defined in DCAT-AP, respectively become Organization, Package/Dataset in the CKAN specification.

Last but not least, to ensure maximum interoperability of the whole system as Open Data, the information (descriptions) provided in CKAN shall be made available in DCAT-AP format, serialised in Resource Description Framework (RDF) documents. This requires a final transformation between the CKAN format and DCAT-AP.

Note that the transformation presented in Table 1 only goes up to the CKAN level due to a module designed in the architecture, as explained in Section 4.3. This module focuses solely on exporting Datasets and Distributions to DCAT-AP.

## 4. NGSI-LD to CKAN Connector

**Figure 1:**
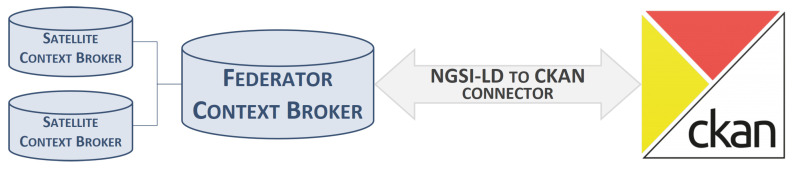
High-level solution setup.

This section presents the proposed solution to address the problem stated in the article: the interworking between the NGSI-LD and the Open Data worlds. Figure 1 shows the baseline scenario including the two key components from each of the two worlds that the implemented connector has to make to interwork. The NGSI-LD world is presented on the left side through a set of CBs in a federation setup, whereas the Open Data world is depicted on the right side via a CKAN instance. The solution proposed takes place between these two components, along with some other processes needed in order to automate the whole pipeline. These additional modules will be further detailed below.

As it was presented in Section 3 the communication between a CB and an Open Data platform as CKAN is not straightforward, since the CB stores actual data while the Open Data Portal stores the description of the datasets. Therefore, the main role of the connector that has been designed and implemented is to create the descriptions of the datasets available through the CB, so that CKAN is able to consume these descriptions. The sought-after full connection between these two paradigms, NGSI-LD and Open Data, is then ensured by the components and processes of this architecture.

To this end, we have defined a three-stepped process: firstly, an understandable description of the data for the CKAN to consume has to be generated; secondly, a harvesting process that imports the descriptions into the CKAN instance has to be triggered; and finally, it is necessary to perform the RDF-compliant serialisation for the generated descriptions to be consumed by the CKAN-based portals.

### 4.1. Phase 1-Creation of Descriptions

**Figure 2:**
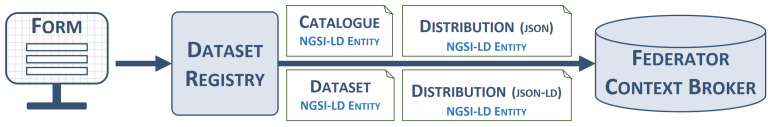
Component architecture of the initial stage.

The initial stage entails the representation of the data. Figure 2 represents the components involved in this first phase, comprising the connection between the user and the FCB. That is, the creation of comprehensive descriptions of the data.

These descriptions are represented using the DCAT-AP subject [24] from the Smart Data Models initiative [13], more specifically Catalogue, Dataset and Distribution data models as discussed in Section 3. By means of these three terms a comprehensive description of a data collection can be made, ranging from the proprietary organisation (Catalogue), through the description of the dataset itself (Dataset), and finally to the physical representations in different formats of the access to the data in question (Distributions).

The approach followed for the definition of datasets involves grouping data (NGSI-LD entities) by entity type (property type). Additionally, two Distributions per Dataset are provided. One using raw JSON format, and the second one employing its linked-data flavour (i.e., JSON for Linked Data (JSON-LD)). Thus, the proposal is to have a Catalogue that identifies the FCB owner, a Dataset for each data type, and two Distributions for each Dataset. According to the DCAT-AP guidelines [8], customisation of numerous parameters requires domain knowledge, including descriptions of the elements themselves, location (e.g., Spain, France) and dataset themes (e.g., Environment, Energy, Health). It is strongly recommended that these parameters take one of the available values of the EU Vocabularies in [25], so that they have standard values defined by an external and reliable institution.

Taking this into account, a web-based form (cf. Figure 2) has been developed as the first element of this initial stage. The aim of this form is to facilitate to the user the generation of descriptions including these customisable attributes. Fields implicated are listed below:

- Type: https://smartdatamodels.org/dataModel.*<subject>*/*<type>*
- Description: *<dataset full description>*
- Creator: *<comma-separated list of entities owning or sources of the data>*
- Provider: *<comma-separated list of entities publishing data>*
- Data Type Topic: *<checkbox list with multiple choice (e.g., Environment, Energy, Health)>*
- Access Rights: *<dropdown list (Publich, Restricted, Private)>*
- Language: *<dropdown list (English, Spanish, German, French)>*
- Location: *<checkbox list with multiple choice (e.g., Austria, Portugal, Spain)>*
- Keywords: *<comma-separated list of related concepts>*

The next component involved in the architecture is the Dataset Registry module, whose source code is available in [26]. This module generates the NGSI-LD entities and injects them into the FCB, which is the last element depicted in the initial stage architecture. More precisely, the Dataset Registry module is responsible for performing the transformation between the output of the form (plain text or JSON, depending on the design) and the defined NGSI-LD entities. Such transformation exploits the principles of linked data provided by the NGSI-LD standard, building relationships between the different entities to generate the tree structure inherent in DCAT-AP. Once these entities are generated, in this case four of them (Catalogue, Dataset, Distribution with JSON format and Distribution with JSON-LD format), the component injects them into the FCB, making them available from the platform’s single access and ready for the next step in the chain.

In addition to the fields that directly convey the user’s information through the form, there exist certain parameters within the Distributions data models that require some further elaboration. These parameters pertain to the Uniform Resource Locator (URL) that enable access to and downloading of the described information. As mentioned earlier, the approach taken for clustering data is based on data type (or entity type), therefore the straightforward method of access via the FCB through the NGSI-LD API [4] would be with the resource /ngsi-ld/v1/entities/?type=<entity_type>. This resource provides all entities of type <entity_type>, in line with the Dataset creation strategy. However, rather than the final value of these parameters (accessUrl and downloadURL) being direct links to the context broker, as described in the section below, an intermediate module has been developed as a proxy that is responsible for redirecting the requests.

### 4.2. Phase 2-Publication of Data in CKAN Instance

The previous section left the platform in the following state: entities Catalogue, Dataset and Distributions are stored in the FCB, all describing the information stored in the federated CBs. Therefore, the next step is the transition to Open Data, for which a CKAN instance is employed. This CKAN instance provides a standard representation of the information through a widespread open data portal that is managed by the overall platform owner.

**Figure 3:**
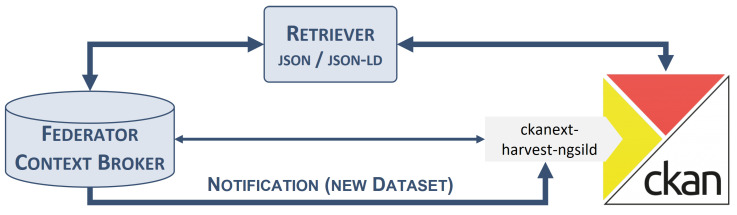
Component architecture of the second stage.

Figure 3 depicts an overview of the components involved in this second phase as well as the interaction between them. As can be seen, the platform includes the FCB, a CKAN instance with a submodule as an extension, and the Retriever module, which will be discussed later.

Focusing on the CKAN submodule, it has been already mentioned in Section 2 that CKAN allows the development of extensions, so that its functionality and features can be extended for the convenience of the user [27]. Taking this into account, this extension submodule called ckanext-harvest-ngsild has been developed (source code available at [28]), and is responsible for harvesting the description entities (Catalogue, Dataset and Distribution) stored in the FCB and injecting them into the deployed CKAN instance.

The extension module subscribes to Dataset entities in the FCB, so that each time a new entity is injected through the Dataset Registry module a notification containing that entity is received. By means of this information, ckanext-harvest-ngsild transforms both the Catalogue and Dataset, as well as the associated Distributions from Smart Data Models into the corresponding CKAN format (organisation, package and resources) following the mapping presented in Table 1, Table 2 and Table 3, and injects them into the CKAN portal. As discussed at the end of the previous section, some properties as accessUrl and downloadURL point to the endpoints made accessible by an intermediate module, the so-called Retriever in Figure 3. This module is intended to provide the data stored in the federated CBs and available through the FCB in different formats. Therefore, not only does the Retriever module act as a reverse proxy but it also transforms the data into multiple representations. The CKAN extension provides three additional endpoints to the CKAN portal URL. These are:

- /ngsi-ld/subscribe: this endpoint aims to create the subscription to the FCB that has just been explained. This subscription, that follows the entity pattern defined in [4], includes the /ngsi-ld/notifications endpoint as the callback for the notifications.
- /ngsi-ld/unsusbcribe: this endpoint is responsible for unsubscribing from the FCB, stopping the reception of notifications triggered by the registration of new entities of type Dataset.
- /ngsi-ld/notifications: this endpoints corresponds to the URL resource that receives the notifications from the FCB to which the module is subscribed. When a notification arrives, it triggers the transformation to CKAN format and the creation (or update) of the package (dataset) together with its resources (distributions) in the CKAN portal.

It is worth noting that these endpoints are restricted to the system administrator, due to the necessity of specific parameters in the requests. These parameters refer to the CKAN user performing the request along with an API Token to authenticate against the CKAN platform.

As discussed previously, two Distribution entities are generated for each Dataset, describing the access to the data in JSON and JSON-LD format respectively. It has been highlighted that the accessUrl and downloadURL fields are essential to the architecture as they specify the URL for accessing and downloading the described data. This URL typically points directly to the resources offered by the FCB, but due to limitations in the use of headers required when making requests, it is necessary to generate an intermediate module, called the Retriever. One of these limitations refers to the representation formats. The FCB can provide the stored data in two formats: JSON and JSON-LD. To enable this option, the HyperText Transfer Protocol (HTTP) header Accept (i.e., Accept: application/json) must be specified. Given this aspect, the Retriever module is developed (source code available at [29]) and incorporates two endpoints:

- /retriever/realtime/__<url_type>__.<format>: this endpoint is intended for real time data requests, i.e., the last instance recorded in the CB. The <url_type> field refers to the complete URL path that describes the type of entity being requested and the <format> field refers to the format in which information has to be retrieved. For example, /retriever/realtime/__https://smartdatamodels.org/dataModel.Parking/ParkingSpot__.jsonld will provide the latest values recorded for the entity type ParkingSpot that belongs to the Parking subject [30] in JSON-LD format.
- /retriever/temporal/__<url_type>__.<format>?<temporal_unit>=<value>, where temporal_unit = ["year", "months", "weeks", "days", "hours"]: this endpoint allows to perform requests with temporal context, i.e., making use of the temporal storage provided by the CB. The <url_type> and <format> fields remain the same as in the previous scenario. Nonetheless, there exist the possibility of adding a query parameter, <temporal_unit>, which indicates the time unit to be used, and, its value (<value>). The possibilities currently deployed for the time unit are those discussed above: years, months, weeks, days and hours. For example, /retriever/temporal/__https://smartdatamodels.org/dataModel.Parking/ParkingSpot__.json?days=5 will provide the stored values of the last 5 days for the entity type Parking/ParkingSpot [30] in JSON format.

Once the Retriever receives the request, it transfers the necessary parameters to an NGSI-LD Query to obtain the values demanded in the first request. This transformation looks like the following:

- Request sent to Retriever:
  GET /retriever/realtime/__<url_type>__.<format>
- Request generated internally in Retriever and sent to FCB:
  GET /entities?type=<url_type>
  Accept: application/<format>

Hence, this module is used to supply the URLs for accessing the data, with the appropriate parameters in each individual case. This results in the completion of the Retriever module. Its purpose is twofold: to hide the FCB from the outside world (thus protecting against the possibility of malicious requests such as HTTP POST or PUT), and to allow data to be retrieved in multiple formats by means of Open Data portals.

### 4.3. Phase 3-DCAT Serialisation

This is an additional step towards compliance with the DCAT-AP standard beyond Smart Data Models. Essentially, its purpose is to export the data descriptions stored in CKAN into RDF documents serialised using DCAT. Despite CKAN’s popularity, this approach ensures maximum interoperability. To achieve this objective, a new CKAN extension has been developed to transform the descriptions of Organisations, Datasets and Resources via the mapping relationships shown in Table 2 and Table 3 into RDF documents compliant with DCAT-AP v2.1.1.

**Figure 4:**
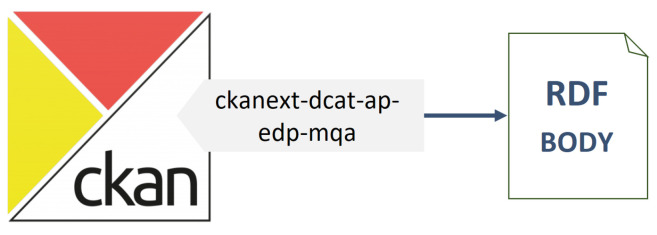
Component architecture of the additional step towards full interoperability.

To this end, the resulting architecture is shown in Figure 4, depicting the new extension developed for CKAN and its output.

This extension, ckanext-dcat-ap-edp-mqa, uses the ckanext-dcat extension [31] as a starting point, which already performs a first transformation between both worlds (CKAN and DCAT-AP). This base extension enables certain endpoints in the CKAN instance, the most important being https://<ckan-instance-host>/dataset/<dataset-id>.<format>. Via this endpoint, <dataset-id> dataset is exported into the specified RDF serialisation format (<format>). The format parameter is compatible with four values: RDF/XML (rdf or xml, application/rdf+xml), Turtle (ttl, text/turtle), Notation3 (n3, text/n3), and JSON-LD (jsonld, application/ld+json). Adding the profiles parameter allows customisation on how values defined in CKAN are mapped to the RDF graph. Moreover, two profiles are provided within this base extension: euro_dcat_ap_2 and euro_dcat_ap. The latter is fully compatible with DCAT-AP v1.1.1, while the former is partially compatible with DCAT-AP v2.1.0. Meanwhile, the extension proposed in this work, ckanext-dcat-ap-edp-mqa, generates a new profile called dcat-ap-edp-mqa which is fully compatible with DCAT-AP v2.1.1, through the transformation presented in Table 2 and Table 3. Thus, using the endpoint shown above by adding the profiles parameter as follows: /dataset/<dataset-id>.<format>?profiles=dcat_ap_edp_mqa, the RDF graph of the dataset dataset-id is obtained, fully compatible with the DCAT-AP v2.1.1 standard. The source code of the ckanext-dcat-ap-edp-mqa extension is available at [32].

## 5. Validation Scenario Implementation

This section outlines the specific use case that has been employed for the implementation and validation of the modules described above. The NGSI-LD-based domain used for the validation was the Data Enrichment Toolchain (DET) implemented and deployed within the Situation-Aware Linked heTerogeneous Enriched Data (SALTED) project [33]. This toolchain harmonises and enriches heterogeneous data by applying the principles of linked data and semantics, with the aim of achieving the so-essential interoperability. Moving on to the CKAN domain, the SALTED project has also deployed a dedicated CKAN instance [34] that represents the data stored in the CBs hosted on the platform. Additionally, the EDP constitutes a final consumer of the data exposed in CKAN [35]. The EDP [36] is the central point of access to European Open Data. It is the preferred tool for European initiatives to publish their data given its main objectives, among which its mission is focused on giving open access, high-quality and available data within the European Union. Therefore, it is a reliable source of information and helps with the open dissemination of data.

**Figure 5:**
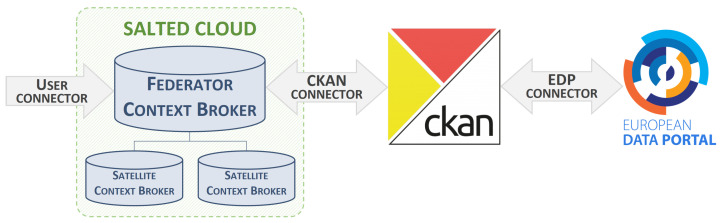
Architecture of the use case with the set of connectors.

Figure 5 illustrates the final architecture of the use case, incorporating the connectors described so far. The key components are the federated architecture of CBs, which exposes the data enriched within the DET deployed in SALTED (and hosted in the SALTED Cloud) through its NGSI-LD API; the SALTED CKAN-based Open Data portal, which describes the datasets available; and finally the EDP with the SALTED data catalogue.

The integration of EDP as a consumer of the information stored in CKAN makes it necessary to meet certain requirements concerning the description of the datasets (Catalogue, Datasets and Distribution entities). The Metadata Quality Assessment (MQA) procedure [37], which the Datasets’ descriptions undergo once they are published in this Open Data portal, sets out these conditions. As the central access point to European data, they ensure that the information published through this organisation is of high quality, or at least that this quality is labelled so that any user seeking to discover and use information is aware of what they are consuming.

These enforced MQA requirements address these aspects [37]: Findability, using metrics that help people and machines find datasets; Accessibility, using metrics that describe whether access to data is guaranteed; Interoperability, using metrics that determine whether a distribution is considered interoperable; Reusability, using metrics that check whether data is reusable; and Contextuality, using metrics that provide more context to the user. Therefore, the MQA process has been considered in the development of the key components.

The first component involved is the form, located within the so-called User Connector, which represents the first phase of the workflow. This form has been designed, as mentioned above, to allow the user to customise all those parameters of the DCAT-AP standard that require some domain knowledge about the data to be described. Likewise, it also sets out to use the default values provided by [25], which are those checked by the EDP in order to guarantee the quality dimensions defined in terms of Data Type Topic, Access Rights, Language and Location (form fields).

The Dataset Registry, which is also located within the User Connector, is the second component concerned. This module takes the information received from the form and generates the Catalogue, Dataset and Distribution entities. Besides integrating this information, this module adds additional details to achieve more complete descriptions and thus score higher in the EDP MQA process.

Moving on to the next stage, there is the CKAN Connector, which brings together the developed extension and the Retriever module. In terms of the latter, the addition of the EDP at the end of the workflow has been taken into account while generating the URL resources for the data access and download properties, as mentioned above. The HTTP HEAD method has been enabled for the requests received in this module, as the EDP uses this method during the MQA process to check the accessibility of these resources. Concerning the extension of the CKAN instance, this plug-in performs the conversion from NGSI-LD to CKAN format, recalling that this transformation makes use of the mapping relationships shown in Table 1, Table 2 and Table 3. The existence of the EDP as a consuming entity does not alter its functionality in this case, since the NGSI-LD entities are already completed at DCAT-AP level.

**Figure 6:**
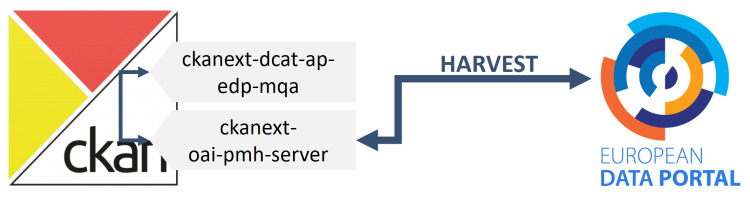
Component architecture of the EDP connector.

The last phase involves the EDP connector, which was partially covered in the previous section with the extension ckanext-dcat-ap-edp-mqa. The behaviour of this extension is verified thanks to the Shapes Constraint Language (SHACL) service provided by data.europa.eu [38], which performs a validation of the RDF document body. However, an additional extension has been developed to ensure that the CKAN-stored information is understandable to the EDP, by creating a resource to which the EDP can make requests and consume the DCAT-AP descriptions provided. Figure 6 presents the architecture of the EDP connector, showing the already explained ckanext-dcat-ap-edp-mqa extension along with a new one called ckanext-oai-pmh-server. The source code of this new extension is available at [39]. According to the EDP documentation [40], its current version of the harvester supports harvesting from an Open Archives Initiative Protocol for Metadata Harvesting (OAI-PMH) [41] compliant source. Consequently, in this final extension, the RDF descriptions of the Datasets and Distributions are available through the usage of this protocol.

Following the OAI-PMH documentation [41] in combination with the EDP [40], the latter says that it only makes use of the ListRecords verb. This verb is designed to harvest records from a repository. In other words, it returns a list of records stored in a repository, in our case all those Catalogues, Datasets and Distributions available [41]. The corresponding URL looks like this: https://<ckan-instance-host>/oai?verb=ListRecords&metadataPrefix=<metadataPrefix>. In successive requests the resumptionToken parameter must be used, in order to keep track of the elements in the list.

Thanks to this extension, the EDP can now access the data stored in the CKAN and publish it in an open and accessible way. The SALTED Project catalogue in the EDP can be seen in [42].

**Figure 7:**
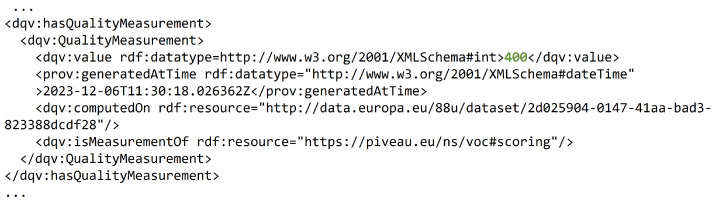
Extract from DQV file about the Parking:ParkingSpot dataset [44]. Scoring is highlighted in green.

Quality metrics for a dataset that has undergone the complete workflow are provided in [43] and illustrated in Figure 7. In [43] it can be seen that the dataset (Parking:ParkingSpot in particular) scores highly in all metrics except ByteSize, since it was not possible to determine the total size of the dataset due to a design constraint that defined the datasets as real-time values. According to the EDP documentation [37], 405 points is the maximum that can be achieved. In this particular case, in the absence of the points related to the ByteSize property, a total of 400 points is obtained, as can be seen in Figure 7. This file extract is sourced from the Data Quality Vocabulary (DQV) file [44], encompassing values for each metric and the overall points computation.

Based on these results, it is clear that the proposed connector architecture achieves the established goals. Starting with the deployment of a CKAN instance as an open data portal for the SALTED project. This CKAN instance is automatically fed through the proposed architecture, so that the open data portal stores data descriptions (Catalogue, Dataset and Distribution) in a way that is compatible with the infrastructure. The second major result is demonstrated by the interoperability achieved with DCAT-AP from the CKAN instance itself. For the third significant result, there is the connection between the CKAN instance of the project and the EDP, implementing the appropriate interface so that the EDP can successfully consume the data, publishing the SALTED catalogue on its well-known platform. And finally, the fourth milestone is achieved through the EDP’s MQA process score obtained in each of the published datasets as well as at the catalogue level, placing the project and its open data within the Excellent ranking.

## 6. Conclusions

Both NGSI-LD-based and CKAN-based Open Data are domains constantly expanding and growing nowadays, revealing the potential benefit and opportunity for bridging the gap between them. The synergy between the Open Data portals’ ability to describe datasets and the NGSI-LD’s proficiency in providing the actual data through an interoperable data model not only increases the volume of publicly available information, but also widens the potential for significant analysis and empowered decision making.

In this paper we have presented the work carried out to develop a solution consisting of a set of connectors aimed at establishing a real connection between the two domains. The technological contributions of this work comprise:

- The analysis of both domains, finding out the ways to integrate them.
- The analysis of the data models and representation formats encountered throughout the entire pipeline.
- The design and development of modules and mechanisms for the completion of the proposed architecture.
- The integration of these modules within an established platform in order to validate the toolchain proposed.
- The integration of these modules with the widely known EDP in order to enrich the depth of the public information available through this portal.

All in all, the proposed architecture consists of nine components: Form, to enable user interaction when describing datasets; Dataset Registry, to perform the transformation of form input to NGSI-LD; Context Brokers architecture, for the storage and provision of context information; CKAN, as the project’s/initiative’s personal open data portal; three extensions to CKAN, to extend the functionalities of that platform in terms of data injection, data export in different formats and provision of an interface for data consumption; Retriever, to hide the Context Brokers architecture from the outside; and finally, the EDP, as the final element for data consumption and publication in its widely acknowledged open data portal.

Possible extensions of this work are, firstly, pertaining to the developed Retriever module, to enlarge its functionality in order to allow more representation formats such as XML syntax for RDF (RDF/XML), Turtle or Notation3. This leads to the second future idea, which is to modify the Dataset Registry to generate Distributions not only in JSON and JSON-LD, but also in the rest of these formats supported by the Retriever. On the other hand, another possible future evolution of the connector is to avoid the need for deploying the CKAN instance, and directly bridge any NGSI-LD platform with the EDP without such an intermediate domain. Nevertheless, this would require a thorough comparison between the two alternatives, both with and without CKAN, to determine which is the most appropriate. In this paper, the CKAN instance has been used due to the fact that the vast majority of current Open Data cases are based on this framework, allowing each organisation to have its own Open Data portal, regardless of the fact that they may later be aggregated into a broader one such as the EDP. This is, precisely, why we opted for this alternative, as it allows for a more generic approach.

## Figures and Tables

**Table 1:** DCAT-AP/Catalogue Smart Data Model conversion to CKAN format.

Smart Data Model	CKAN
**Catalogue**	**Organization**
id	id
title	title
	name
description	description
homepage	extras:url
publisher	N/A
dataset	packages

**Table 2:** DCAT-AP/Dataset Smart Data Model conversion to CKAN format and DCAT-AP specification.

Smart Data Model	CKAN	DCAT-AP
**Dataset**	**Package**	**dcat:Dataset**
id	id	N/A
description	notes	dct:description
title	title	dct:title
	name	N/A
accessRights	extras:access_rights	dct:accessRights
creator	author	N/A
distribution	resources	dcat:distribution
keyword	tags	dcat:keyword
landingPage	url	dcat:landingPage
language	extras:language	dct:language
license	license_id	N/A
publisher	owner_org	dct:publisher
spatial	extras:spatial	dct:spatial
temporal	extras:temporal_start	dct:temporal
theme	extras:theme	dcat:theme
hasVersion	extras:has_version	N/A
versionNotes	extras:version_notes	owl:versionInfo
dataProvider	maintainer	dcat:contactPoint
dateCreated	metadata_created	N/A
	extras:issued	dct:issued
dateModified	metadata_modified	N/A
	extras:modified	dct:modified

**Table 3:** DCAT-AP/Distribution Smart Data Model conversion to CKAN format and DCAT-AP specification.

Smart Data Model	CKAN	DCAT-AP
**Distribution**	**Resource**	**dcat:Distribution**
description	description	dct:description
title	name	dct:title
accessUrl	url	N/A
	access_url	dcat:accessURL
availability	N/A	N/A
byteSize	size	dcat:byteSize
downloadURL	download_url	dcat:downloadURL
license	license	dct:license
mediaType	mimetype	dcat:mediaType
rights	rights	dct:rights
dateCreated	created	dct:issued
dateModified	last_modified	dct:modified
format	format	dct:format

## Data Availability

No new data were created or analyzed in this study. Data sharing is not applicable to this article.

## Abbreviations

The following abbreviations are used in this manuscript:

API	Application Programming Interface
CB	Context Broker
CKAN	Comprehensive Knowledge Archive Network
DCAT	Data Catalog Vocabulary
DCAT-AP	Data Catalog Vocabulary-Application Profile
DET	Data Enrichment Toolchain
DMS	Data Management System
DQV	Data Quality Vocabulary
EDP	European Data Portal
FAIR	Findability, Accessibility, Interoperability and Reusability
FCB	Federator Context Broker
HTTP	Hypertext Transfer Protocol
ICT	Information and Communication Technology
IoT	Internet of Things
JSON	JavaScript Object Notation
JSON-LD	JavaScript Object Notation for Linked Data
MQA	Metadata Quality Assessment
NGSI-LD	Next Generation Service Interface with Linked Data
OAI-PMH	Open Archives Initiative Protocol for Metadata Harvesting
RDF	Resource Description Framework
RDF/XML	XML Syntax for RDF
SALTED	Situation-Aware Linked heTerogeneous Enriched Data
SHACL	Shapes Constraint Language
URL	Uniform Resource Locator
W3C	World Wide Web Consortium
